# Buyer beware? Does the information provided with herbal products available over the counter enable safe use?

**DOI:** 10.1186/1741-7015-9-94

**Published:** 2011-08-09

**Authors:** David K Raynor, Rebecca Dickinson, Peter Knapp, Andrew F Long, Donald J Nicolson

**Affiliations:** 1 School of Healthcare, University of Leeds, Woodhouse Lane, Leeds, LS2 9UT, UK; 2 Department of Health Sciences, University of York, YO10 5DD, UK; 3 Quality, Safety and Informatics, Clinical and Population Sciences & Education Division, University of Dundee, Ninewells Avenue, Dundee, DD1 9SY, UK; 4 Current address: Health Sciences, Warwick Medical School, Coventry CV4 7AL, UK

## Abstract

**Background:**

Herbal products obtained over the counter are commonly used in Europe, North America and Australia. Although there is concern about a lack of information provided to consumers to allow the safe use of these products, there has been no published research to confirm these fears. In this study, we evaluated written information provided with commonly used herbal products in the UK in advance of a European Union Directive issued in April 2011 that tightened regulations for some herbal products, including requirements to provide safety information.

**Methods:**

Five commonly used herbal products were purchased from pharmacies, health food shops and supermarkets: St John's wort, Asian ginseng, echinacea, garlic and ginkgo. Written information provided with the products (on the package or on a leaflet contained in the package) was evaluated for inclusion of each of the key safety messages included in the monographs of the US National Center for Complementary and Alternative Medicine. Specifically, we looked for information on precautions (such as Asian ginseng not being suitable for people with diabetes), interactions with conventional medicines (such as St John's wort with the contraceptive pill and warfarin) and side effects (such as ginkgo and allergic reactions).

**Results:**

Our analysis showed that, overall, 51 (75%) of 68 products contained none of the key safety messages. This included 4 of 12 St John's wort products, 12 of 12 ginkgo products, 6 of 7 Asian ginseng products, 20 of 21 garlic products and 9 of 13 echinacea products. The two products purchased that are registered under the new European Union regulations (for St John's wort) contained at least 85% of the safety messages.

**Conclusions:**

Most of the herbal medicine products studied did not provide key safety information which consumers need for their safe use. The new European Union legislation should ensure that St John's wort and echinacea products will include the previously missing information in due course. The legislation does not apply to existing stock. Depending on therapeutic claims made by manufacturers, garlic, ginkgo and Asian ginseng products may not be covered by the legislation and can continue to be bought without the safety information. Also, consumers will still be able to buy products over the internet from locations outside European Union jurisdiction. Potential purchasers need to know, in both the short term and the long term, how to purchase herbal products which provide the information they need for the safe use of these products.

## Background

Complementary and alternative medicines (CAM) are now mainstream in the UK and the rest of Europe, as well as in North America and Australia [1-4]. Consumers of herbal products available over the counter need access to reliable and readily accessible information to ensure their safe and appropriate use. The written information people get when they buy herbal products is particularly important because, although research in this area is sparse, it has been shown that in the UK staff knowledge about the products sold in community pharmacies and health food shops can be lacking [5].

Research carried out for the UK medicines regulator, the Medicines and Healthcare products Regulatory Agency (MHRA) [1], found that many people believe that herbal products are safe because they are natural, confirming previous findings [6,7], and that patients often refrain from telling their physicians if they are using an herbal product. Equally important is that few doctors ask patients about their use of CAM, including herbal products [8].

Many herbal products have adverse effects that are not acknowledged or known by their users, with about one-third being unaware of any possible risks [9]. In addition, most healthcare professionals believe that the public is poorly informed about herbal products [10]. A report by the MHRA [11] stated, *'With typical western herbal medicine e.g. found in health food shops supermarkets etc the most frequent area of concern in the unlicensed sector is lack of systematic patient information' *(p. 6).

The need for reliable information is one of four points highlighted in the World Health Organisation (WHO) Traditional Medicines Strategy of 2002 [12]. In addition, a WHO World Health Assembly resolution issued in 2003 urged member states to provide reliable information to consumers to promote proper use of CAM.

Key pieces of information about the safe use of herbal products that consumers need to know include (1) precautions, because many herbal products can be unsafe for use by people with some preexisting illnesses, for example, the use of Asian ginseng by people with diabetes; (2) interactions with other products, for example, the use of St John's wort affecting the efficacy of the contraceptive pill or warfarin; and (3) adverse effects, for example, allergic reactions associated with the use of ginkgo [13]. Without such information, it will be more difficult for the consumer to be able to make an informed choice whether to use CAM, with possible consequent adverse effects.

In some European countries, the use of herbal remedies is widespread and well established [14]. Other countries have a historical tradition of using herbal medicines, such as the UK [15], and in other European countries there are existing regulations for CAM (for example, Austria and Germany [3]). Until 2011 in the UK, there were three possible regulatory routes by which an herbal product could reach a consumer. The most common route was an unlicensed herbal remedy, which does not have to meet specific standards of safety and quality and is not required to be accompanied by safety information for the consumer [11]. To help the public make informed choices about the use of herbal products, a European Union (EU) directive was implemented in April 2011 after a seven-year transition period. The goal of this directive is to harmonise the regulation of traditional herbal medicine products across the EU and to establish a simplified licensing system [16]. It requires that all manufactured herbal products either gain a product licence of the type needed to manufacture 'conventional' products or become registered as a 'traditional herbal medicinal product'.

### Licensed herbal medicines

Licensed herbal medicines hold a product licence, as do conventional medicines (that is, based on safety, quality and efficacy), and must be accompanied by comprehensive information for the patient regarding their safe use. This information comprises six categories: indications, precautions, how to use the product, side effects, how to store the product and regulatory information, all of which are usually provided on a leaflet inserted into the product package [17]. In addition, the leaflets have to be tested with lay people to ensure that they are clear and understandable [18]. However, it is widely recognised that, although there is some evidence of efficacy for some herbal medicines, evidence of reproducible efficacy in many cases is currently insufficient to meet regulatory standards. Hence a licence to sell these products cannot be obtained.

### Registered traditional herbal medicine

The new category of Traditional Herbal Registration (THR) was created in April 2004 with a transition period of seven years [19]. As a result, in the UK, a 'simplified registration scheme', the Traditional Herbal Medicines Registration Scheme, was introduced. In this scheme, herbal medicine products have been required to meet specific standards of safety and quality, have agreed-upon indications for use based on their traditional use and include information for the purchaser to promote the safe use of the product, which is provided to the consumer in the form of a leaflet similar to that required for licensed products [17]. The resulting deadline was 30 April 2011.

We found no previous studies on patient information supplied with herbal products available for purchase in the UK. Building on previous work describing consumer information provided with conventional medicines [20], we aimed to evaluate the information provided with a select set of common herbal products available for purchase in the UK. The objective was to ascertain whether the products contained the minimum information required to enable their safe use.

## Methods

### Study design

We conducted a cross-sectional survey involving content analysis of written information provided with five commonly used herbal products: St John's wort, garlic, ginkgo, Asian ginseng and echinacea. These herbal products were chosen because one or more of the following factors applied to each of them: evidence of interaction between herbal medicine and a prescribed medicine [21], a risk-benefit profile had been undertaken [22] and availability in retail outlets in local shopping areas. We chose to include single-ingredient products intended for oral use. We excluded products available as creams, liquids, oils, sprays, teas and tinctures.

### Obtaining the products

In the UK, herbal remedies are available in health food shops and mainstream outlets such as pharmacies and supermarkets [23,24]. Hence we bought all of the oral use products containing one of the five herbal products from three types of retail establishment in one city. The sources were (1) two health food stores, Holland and Barrett (the largest UK retail chain in this sector), and an independent health food store associated with the National Association of Health Food Stores; (2) three pharmacies based in supermarkets (Tesco, Sainsbury's and Asda); and three large chain pharmacies (Boots, Superdrug and Lloyds Chemists).

### Evaluation criteria

We evaluated the information that accompanies each product for completeness and accuracy in the key safety areas of precautions, interactions and side effects. The assessment of the completeness and accuracy of the information was not straightforward. There is no single, recognised, authoritative source of information on herbal medicines. Reference texts for herbal medicines include Barnes *et al. *[25], the Natural Medicines Comprehensive Database [26], Mills and Bone [27], Blumenthal *et al. *[28], the European Medicines Agency [29] and the US National Center for Complementary and Alternative Medicine (NCCAM) [13].

However, no single text appears to be regarded as the gold standard. In addition, the evidence basis for some precautions, interactions and side effects is limited, and sometimes there is no consensus regarding what these are and their level of significance.

As this study was a general exercise designed to broadly determine the appropriateness of information provided with herbal medicine products, we chose to base it on data contained in a free access resource containing the most important and widely accepted safety information. The Community Herbal Monographs published online by the European Medicines Agency [29] did not cover all five herbal medicine preparations. However, they are all covered by the monographs produced by the NCCAM [13], which were therefore chosen. NCCAM is part of the US National Institutes of Health, and one of its roles is "disseminating authoritative information to the public and professionals". The monographs are short (two pages) and contain only the most important information and warnings.

Each monograph was analysed for content to identify information related to precautions, interactions and side effects. Analysis of the NCCAM monographs yielded 16 points of information for St John's wort, 11 for ginkgo, 8 for garlic and for Asian ginseng, and 6 for echinacea (Table 1).

**Table 1:** Key safety issues^a^

St John's wort	Ginkgo	Asian ginseng	Garlic	Echinacea
**Precautions**				
Sensitivity to sunlight	Increased bleeding risk	People with diabetes should use extra caution, especially if using medicines to lower blood sugar or taking other herbs ... also thought to lower blood sugar.	Can thin the blood	Allergy to related plants in daisy family
	Bleeding disorders		Use with caution if planning to have surgery or dental work.	People with asthma or atopy may be more likely to have an allergic reaction.
	Scheduled surgery or dental procedures		Use with caution if you have a bleeding disorder.	
**Interactions**				
Antidepressants	Anticoagulant drugs		Interferes with effectiveness of saquinavir, a drug used to treat HIV	
Oral contraceptives				
Digoxin				
Indinavir and possibly other HIV medications				
Irinotecan and possibly other anticancer drugs				
Seizure control drugs				
Warfarin and related anticoagulants				
Cyclosporine				
**Side effects**				
Anxiety	Headache	Headache	Breath and body odour	Allergic reactions including rashes
Dry mouth	Nausea	Sleep problems	Heartburn	Increased asthma
Dizziness	Gastrointestinal upset	Gastrointestinal problems	Upset stomach	Anaphylaxis
Gastrointestinal symptoms	Diarrhoea	Allergic reactions	Allergic reactions	Gastrointestinal side effects
Fatigue	Dizziness	Breast tenderness		
Headache	Allergic skin reactions	Menstrual irregularities		
Sexual dysfunction	More severe allergic reactions occasionally reported	High blood pressure		

^a^As identified in the NCCAM Herbs at a Glance monographs [13].

We then performed a structured content analysis of the information supplied with each of the purchased products on the outer and inner packaging and any leaflet contained within the package. Each piece of information was marked as being 'present and accurate' or 'inaccurate or absent'. We marked information as correct if it broadly described the information correctly. For example, where the interacting medicine was digoxin, a leaflet description of 'some products for heart disease' was deemed to be accurate.

### Data extraction and quality assurance

All of the textual information on each product container (and leaflet, if present) was photocopied so that we could create a master copy of the information supplied with each product. This information was then examined by one researcher (RD), and the presence or absence of each point of information was entered into a Microsoft Excel database (Microsoft, Redmond, WA, USA). The nine products supplied with an information leaflet were also assessed by a second researcher (DKR). A check of a randomly selected 10% of the products with information on the package only was independently conducted (DKR). Any differences were resolved by discussion to reach consensus.

## Results

### Nature of the products

We found 68 products sold by 8 different retailers which met the inclusion criteria: garlic, 21; St John's wort, 15; echinacea, 13; ginkgo, 12; and Asian ginseng, 7.

### Regulatory category

The great majority (93%) of products were unlicensed. Of these, 48 stated that they were food supplements. The rest had no classification stated, so the actual product classification could only be determined on a case-by-case basis by the regulator. It is likely that a number of products were supplied as unlicensed herbal medicines under the Medicines Act 1968. The remaining five products (7%) had either a product licence or THR. The breakdown of the herbal products was as follows: unlicensed, 63; THR, 2; and licensed product, 3.

### Information provided

Among the 68 products, 59 (87%) of the manufacturers provided information on the package and/or on the label only, and nine also provided a leaflet package insert. The latter nine products were St John's wort (4 of 15), echinacea (3 of 13) and garlic (2 of 21). Each of the products with a product licence (*n *= 3) or THR (*n *= 2) had a leaflet, but the remainder were unlicensed. One of these leaflets mostly contained information promoting other products. However, as the leaflet contained some brief information on the herbal medicine concerned, it was included.

### Key points of safety information

Tables 2, 3, 4, 5 and 6 list each product by the number allocated to it in this study ('study number'), its legal category (where stated on the product), whether a leaflet was supplied with the product and the number of safety messages included (sub-divided into 'Precautions', 'Interactions' and 'Side effects'). The two products with THR both contained 14 of 16 of the points of information. Table 7 lists the number of products which contained none of the points of information.

**Table 2:** St John's wort^a^

Study number	1	2	3	4	67	70	72	75	30	31	32	41	48	58	61
Where bought	HF	HF	HF	HF	HF	HF	HF	HF	Ph	Ph	Ph	Ph	Ph	SM	SM
Legal category	U	U	U	THR	U	U	U	U	U	THR	U	U	U	U	U
Leaflet supplied?	No	No	No	Yes	No	No	No	No	No	Yes	Yes	No	Yes	No	No
Precautions, *n *= 1	1	0	1	1	0	0	0	1	1	1	1	0	1	0	1
Interactions, *n *= 8	0	0	0	8	0	0	0	0	7	8	7	0	7	0	0
Side effects, *n *= 7	0	0	0	5	0	0	0	0	0	5	0	0	0	0	0

^a^HF = health food shop, Ph = pharmacy, SM = supermarket, U = unlicensed, THR = traditional herbal registration, PL = product licence. Each product is listed by study number, where St John's wort was bought, legal category, whether a leaflet was supplied and the number of safety messages (under 'Precautions', 'Interactions' and 'Side effects').

**Table 3:** Ginkgo^a^

Study number	5	6	7	8	73	33	34	35	36	42	56	64
Where bought	HF	HF	HF	HF	HF	Ph	Ph	Ph	Ph	Ph	SM	SM
Legal category	U	U	U	U	U	U	U	U	U	U	U	U
Leaflet supplied?	No	No	No	No	No	No	No	No	No	No	No	No
Precautions, *n *= 4	0	0	0	0	0	0	0	0	0	0	0	0
Side effects, *n *= 7	0	0	0	0	0	0	0	0	0	0	0	0

^a^HF = health food shop, Ph = pharmacy, SM = supermarket, U = unlicensed.

**Table 4:** Asian ginseng^a^

Study number	9	10	12	15	76	55	60
Where bought	HF	HF	HF	HF	HF	SM	SM
Legal category	U	U	U	U	U	U	U
Leaflet supplied?	No	No	No	No	No	No	No
Precautions, *n *= 1	0	0	1	0	0	0	0
Side effects, *n *= 7	0	0	0	0	0	0	0

^a^HF = health food shop, SM = supermarket, U = unlicensed.

**Table 5:** Garlic^a^

Study number	19	20	21	22	23	24	25	26	69	71	74	43	49	50	52	53	54	45	46	59	62
Where bought	HF	HF	HF	HF	HF	HF	HF	HF	HF	HF	HF	Ph	Ph	Ph	Ph	Ph	Ph	SM	SM	SM	SM
Legal category	U	U	U	U	U	U	U	U	U	U	U	U	U	U	U	U	U	U	U	U	U
Leaflet supplied?	No	No	No	No	Yes	No	No	No	No	No	No	No	No	No	No	No	Yes	No	No	No	No
Precautions, *n *= 4	0	0	0	0	0	0	0	0	0	0	0	0	0	1	0	0	0	0	0	0	0
Side effects, *n *= 4	0	0	0	0	0	0	0	0	0	0	0	0	0	0	0	0	0	0	0	0	0

^a^HF = health food shop, Ph = pharmacy, SM = supermarket, U = unlicensed.

**Table 6:** Echinacea

Study number	66	68	77	16	17	37	38	39	40	47	57	44	63
Bought from	HF	HF	HF	HF	HF	Ph	Ph	Ph	Ph	Ph	SM	SM	SM
Legal category	U	U	U	PL	U	PL	U	U	U	PL	U	U	U
Leaflet supplied?	No	No	No	Yes	No	Yes	No	No	No	Yes	No	No	No
Precautions, *n *= 2	0	0	1	0	1	0	0	0	0	0	0	0	0
Side effects, *n *= 4	0	0	0	1	0	1	0	0	0	0	0	0	0

^a^HF = health food shop, Ph = pharmacy, SM = supermarket, U = unlicensed, PL = product licence.

**Table 7:** Presence or absence of information points for each category of herbal product

Herbal product	Some points of information	No points of information
St John's wort, *n *= 15 (%)	11 (73%)	4 (27%)
Ginkgo, *n *= 12 (%)	0 (0%)	12 (100%)
Asian ginseng, *n *= 7 (%)	1 (14%)	6 (86%)
Garlic, *n *= 21 (%)	1 (5%)	20 (95%)
Echinacea, *n *= 13 (%)	4 (31%)	9 (69%)
Total, *n *= 68 (%)	17 (25%)	51 (75%)

## Discussion

The public should be able to expect full disclosure of key safety information when they purchase products herbal medicines over the counter [16]. This study has shown that, among five herbal products commonly purchased over the counter in the UK, most contained little or no information regarding what to check for in order to avoid harm before using the product. Three-quarters of the preparations contained no safety information, and only three contained most or all of the points of information studied. For St John's wort, which has generated the most publicity regarding safe use, two-thirds of the preparations failed to mention any of the possible interactions with conventional medicines such as the oral contraceptive or warfarin. This is consistent with the findings of Clauson *et al. *[30] in the USA, where the manufacturers of the vast majority of St John's wort preparations failed to adequately address clinically relevant safety issues.

This study provides evidence supporting the need to strengthen regulation of herbal medicines in the EU [23]. Our results show that where a product (here, St John's wort) was registered as a THR, over 85% of the safety information studied was included in the information for the consumer. That this figure was not 100% reflects, as stated above, the lack of consensus on the safety information associated with individual herbal medicines. The information provided with these THR products reflects the MHRA's assessment and is consistent with data in the European Medicines Agency's Community Herbal Monographs [29].

The number of THR remains small; in this study, the majority of products were not registered, and by April 2011 the MHRA had received 211 applications for THR, among which 104 applications had so far been granted and the remainder were under assessment (R Woodfield, personal communication).

The introduction of the THR regulations in the UK in April 2011 does not mean, however, that the issues highlighted by this research will be fully resolved. First, although most products containing either St John's wort or echinacea are likely to be classified as medicinal products (and hence their manufacturers will be required to provide comprehensive information to the patient), the position of garlic, ginkgo and ginseng is less clear. Those products which make no claims to treat or prevent disease will fall outside the regulatory remit (R Woodfield, personal communication). Second, although unregulated products should no longer be entering the supply chain, stock already legally held by retailers can continue to be sold, although this will be a time-limited issue. Finally, there is a continuing likelihood of internet availability of products from manufacturers and suppliers in countries outside the jurisdiction of the MHRA.

Printing herbal medicine product information for the consumer on the outside of the package is important to enabling its safe use. Cramer *et al. *[5] point to a continued lack of knowledge among pharmacy staff about herbal products available in pharmacies. Furthermore, although research evidence from health food shops was limited, the advice offered there also appears to be varied and inconsistent. Importantly, Cramer and colleagues found that a significant majority of people who buy herbal products need help in selecting an appropriate product. Others have recognised that pharmacists lack training and knowledge regarding herbal remedies [31]. Most pharmacy customers in an Australian study thought pharmacists should provide safety information about herbal products [4], and in Canada, most consumers and pharmacists thought pharmacists should be knowledgeable about natural health products and able to manage possible drug interactions [32].

In addition to the general need for the public to be educated about the safe use of herbal products and know where to find good information about them, there is a related issue about patients telling their healthcare providers about their herbal product use. There appears to be a general reluctance of patients to tell this to their physicians, with one UK study finding that a large majority did not tell their general practitioners about their use of herbal products [33]. Consumers should be encouraged to tell their healthcare practitioners about any use of herbal products, and practitioners should consider initiating such discussions [34]. This does not, however, take away the necessity for accurate and complete written information about precautions, interactions and side effects to be provided to consumers when they buy herbal products.

The most commonly discussed safety problems associated with the use of herbal products relate to interactions with medicine prescribed by a conventional biomedical practitioner. We know that the use of over-the-counter herbal products concomitantly with conventional medicines is widespread and that there are numerous possible interactions [21]. In a study of cardiology patients, among the herbal products singled out, all five included in the present study were described as being especially dangerous in elderly patients [35]. A UK survey of health professionals found that 86% think the public is poorly informed about herbal products, with the main reason for concern being potential interactions with traditional medicines [10].

Apart from the information provided on or in the herbal product package, what other sources do people use to find out about herbal products? Cramer *et al. *[5] found that pharmacy customers accessed information from CAM practitioners, friends and family, books, newspapers, magazines and the internet. However, reliable and readily accessible information sources on herbal medicines are limited [36]. A survey focussing on ginseng, ginkgo and St John's wort found that among 150 websites, 25% contained statements that could cause harm to users of these herbal products and 97% had omitted information [37].

## Conclusions

It is clear that consumers are entitled to better information with the CAM that they buy, so that they are aware of any safety issues and to enable them to make informed decisions [7]. Just as for conventional medicines, this is important for herbal products as well, because harm to the consumer is possible when such information is not available. The 2011 EU legislation will ensure that such information is available for many products, but products not classified as medicinal products will escape the net of regulation. Also, existing stock will continue to be legally available for a period and internet purchases from sources outside the EU will not be covered. This means that consumers need to be advised where and how to purchase herbal products so that the necessary information for safe use will be available to them. They should look for products registered as THR and seek outlets where staff might be anticipated to have knowledge of and training in the safe use of herbal products. In addition, there should be ongoing monitoring of the written information supplied with these products to encourage improvements in this important safety issue.

## Competing interests

DKR is a director of Luto Research, which provides health information writing and testing services.

## Authors' contributions

DKR conceived the study. DKR, RD, PK and AFL developed the study design. DN undertook a literature review to inform the study. RD collected the data. DKR and RD analysed the data. All authors contributed to the drafting of the manuscript and read and gave final approval for its publication.

## Acknowledgements

We thank Richard Woodfield from the MHRA for his advice on regulatory issues and Alison Blenkinsopp for comments on the draft manuscript. The study was funded by the University of Leeds.

## Pre-publication history

The pre-publication history for this paper can be accessed here:

http://www.biomedcentral.com/1741-7015/9/94/prepub

## References

[B1] Public Perceptions of Herbal Medicines http://www.ipsos-mori.com/Assets/Docs/Polls/public-perceptions-of-herbal-medicines-report.pdf

[B2] Committee on the Use of Complementary and Alternative Medicine by the American Public Board on Health Promotion and Disease Prevention Institute of Medicine Complementary and Alternative Medicine in the United States 2005 Washington, DC: The National Academies Press 22379647

[B3] Anquez-Traxler C The legal and regulatory framework of herbal medicinal products in the European Union: a focus on the traditional herbal medicines category Drug Info J 2011 45 15 23

[B4] Braun LA Tiralongo E Wilkinson JM Spitzer O Bailey M Poole S Dooley M Perceptions, use and attitudes of pharmacy customers on complementary medicines and pharmacy practice BMC Complement Altern Med 2010 10 38 10.1186/1472-6882-10-38 20646290 PMC2919443

[B5] Cramer H Shaw A Wye L Weiss M Over-the-counter advice seeking about complementary and alternative medicines (CAM) in community pharmacies and health shops: an ethnographic study Health Soc Care Community 2010 18 41 50 19659947 10.1111/j.1365-2524.2009.00877.x

[B6] Lynch N Berry D Differences in perceived risks and benefits of herbal, over-the-counter conventional, and prescribed conventional, medicines, and the implications of this for the safe and effective use of herbal products Complement Ther Med 2007 15 84 91 10.1016/j.ctim.2006.06.007 17544858

[B7] World Health Organization Guidelines on Developing Consumer Information on Proper Use of Traditional, Complementary and Alternative Medicine 2004 Geneva: World Health Organization

[B8] Gohar F Greenfield SM Beevers DG Lip GYH Jolly K Self-care and adherence to medication: a survey in the hypertension outpatient clinic BMC Complement Altern Med 2008 8 4 10.1186/1472-6882-8-4 18261219 PMC2259297

[B9] Medicines and Healthcare products Research Agency Herbal products: safety update Drug Saf Update 2009 3 1 5

[B10] Anonymous Herbal medicines: what do clinicians know? Drug Ther Bull 2010 48 46 47 20392781 10.1136/dtb.2010.03.0024

[B11] Public Health Risk with Herbal Medicines: An Overview http://www.mhra.gov.uk/home/idcplg?IdcService=GET_FILE&dDocName=CON023163&RevisionSelectionMethod=Latest

[B12] WHO Traditional Medicine Strategy 2002-2005 http://whqlibdoc.who.int/hq/2002/who_edm_trm_2002.1.pdf

[B13] Herbs at a Glance http://nccam.nih.gov/health/herbsataglance.htm

[B14] Calapai G European legislation on herbal medicines: a look into the future Drug Saf 2008 31 428 431 10.2165/00002018-200831050-00009 18422385

[B15] Nissen N Practitioners of Western herbal medicine and their practice in the UK: beginning to sketch the profession Complement Ther Clin Pract 2010 16 181 186 10.1016/j.ctcp.2010.06.001 20920799

[B16] Routledge PA The European Herbal Medicines Directive: could it have saved the lives of Romeo and Juliet? Drug Safety 2008 31 416 418 10.2165/00002018-200831050-00006 18422382

[B17] Herbal Medicines http://www.mhra.gov.uk/Howweregulate/Medicines/Herbalmedicines/index.htm

[B18] Raynor DK Testing, testing: the benefits of user-testing package leaflets Regul Aff Focus 2008 1 16 19

[B19] European Herbal Medicines Directive 2004/24/EC http://eur-lex.europa.eu/LexUriServ/LexUriServ.do?uri=OJ:L:2004:136:0085:0090:en:PDF

[B20] Raynor DK Blenkinsopp A Knapp P Grime J Nicolson DJ Pollock K Dorer G Gilbody S Dickinson D Maule AJ Spoor P A systematic review of quantitative and qualitative research on the role and effectiveness of written information available to patients about individual medicines Health Technol Assess 2007 11 1 160 17280623 10.3310/hta11050

[B21] Izzo AA Ernst E Interactions between herbal medicines and prescribed drugs Drugs 2009 69 1777 1798 10.2165/11317010-000000000-00000 19719333

[B22] Ernst E The risk-benefit profile of commonly used herbal therapies: Ginkgo, St John's Wort, Ginseng, Echinacea, Saw Palmetto, and Kava Ann Intern Med 2002 136 42 53 11777363 10.7326/0003-4819-136-1-200201010-00010

[B23] Quigley G In the pharmacy: integration is interactive J Complement Med 2002 1 44 45

[B24] Collyer F Tovey P The corporatisation and commercialisation of CAM The Mainstreaming of CAM 2004 London: Routledge 198 215

[B25] Barnes J Anderson LA Phillipson JD Herbal Medicines: A Guide for Healthcare Professionals 2002 2 London: Pharmaceutical Press

[B26] Natural Medicines Comprehensive Database http://www.naturaldatabase.com/

[B27] Mills S Bone K The Essential Guide to Herbal Safety 2005 St Louis: Elsevier

[B28] Blumenthal M Busse WR Goldberg A (Eds) The Complete German Commission E Monographs: Therapeutic Guide to Herbal Medicines 1998 Austin, TX: American Botanical Council/Integrative Medicine Communications

[B29] European Medicines Agency: Community Herbal Monographs http://www.ema.europa.eu/ema/index.jsp?curl=pages/regulation/document_listing/document_listing_000212.jsp&murl=menus/regulations/regulations.jsp&mid=WC0b01ac058003380a

[B30] Clauson KA Santamarina ML Rutledge JC Clinically relevant safety issues associated with St John's wort product labels BMC Complement Altern Med 2008 8 42 10.1186/1472-6882-8-42 18637192 PMC2483264

[B31] Houghton P Regulation of herbal medicinal products Pharm J 2009 282 485 486

[B32] Kwan D Boon HS Hirschkorn K Welsh S Jurgens T Eccott L Heschuk S Griener GG Cohen-Kohler JC Exploring consumer and pharmacist views on the professional role of the pharmacist with respect to natural health products BMC Complement Altern Med 2008 8 40 10.1186/1472-6882-8-40 18625059 PMC2483265

[B33] Vickers KA Jolly KB Greenfield SM Herbal medicine: women's views, knowledge and interaction with doctors BMC Complement Altern Med 2006 6 40 10.1186/1472-6882-6-40 17156416 PMC1702550

[B34] Smith M Boon HS Counseling cancer patients about herbal medicine Patient Educ Couns 1999 38 109 120 10.1016/S0738-3991(99)00058-0 14528703

[B35] Tachjian A Maria V Jahangir A Use of herbal products and potential interactions in patients with cardiovascular diseases J Am Coll Cardiol 2010 55 515 525 10.1016/j.jacc.2009.07.074 20152556 PMC2831618

[B36] Gratus C Wilson S Greenfield SM Damery SL Warmington SA Grieve R Steven NM Routledge P The use of herbal medicines by people with cancer BMC Complement Altern Med 2009 9 14 10.1186/1472-6882-9-14 19442268 PMC2685766

[B37] Waiji M Sagaram S Sagaram D Meric-Bernstam F Johnson C Mirza NQ Bernstam EV Efficacy of quality criteria to identify potentially harmful information: a cross-sectional survey of CAM web sites J Med Internet Res 2004 6 21 10.2196/jmir.6.2.e21 PMC1550600 15249270

